# Toothpaste Prevents Debonded Brackets on Erosive Enamel

**DOI:** 10.1155/2015/468582

**Published:** 2015-03-23

**Authors:** Érico Luiz Damasceno Barros, Shelon Cristina Souza Pinto, Alvaro Henrique Borges, Mateus Rodrigues Tonetto, Roger Phillip Ellwood, Ian Pretty, Matheus Coelho Bandéca

**Affiliations:** ^1^CEUMA University, 01 Josué Montello Street, Renascença II, 65075-120 São Luís, MA, Brazil; ^2^University of Ponta Grossa State, General Carlos Cavalcanti 4748, Ponta Grossa, PR, Brazil; ^3^University of Cuiaba, Beira Rio 3100, Jardim Europa, Cuiabá MT, Brazil; ^4^Colgate Palmolive Dental Health Unit, Skelton House Manchester Science Park, Manchester, M15 6SH, UK

## Abstract

This study evaluated the effect of high fluoride dentifrice on the bond strength of brackets after erosive challenge. Eighty-four enamel specimens were divided into seven groups (*n* = 12): WN (distilled water/no acid challenge), W3C (distilled water/3 cycles of acid challenge), and W6C (distilled water/6 cycles of acid challenge) were not submitted to dentifrice treatment. Groups RF3C (regular fluoride dentifrice/3 cycles of acid challenge) and RF6C (regular fluoride dentifrice/6 cycles of acid challenge) were treated with dentifrices containing 1450 *μ*g F^−^/g and HF3C (high fluoride dentifrice/3 cycles of acid challenge) and HF6C (high fluoride dentifrice/6 cycles of acid challenge) were with 5000 *μ*g F^−^/g. Acid challenges were performed for seven days. After bond strength test, there was no significant difference among groups submitted to 3 cycles of acid challenge (*P* > 0.05). Statistically significant difference was found between the regular and high fluoride dentifrices after 6 cycles of acid challenge (<0.05). Similar areas of adhesive remaining were found among control groups and among groups W6C, RF3C, RF6C, HF3C, and HF6C. The high fluoride dentifrice was able to prevent the reduction of bond strength values of brackets submitted to acid challenge. Clinical relevance: the high fluoride toothpaste prevents debonded brackets on erosive enamel.

## 1. Introduction

Many factors may influence the retention of brackets during orthodontic treatment with fixed appliances [1]. These include the quality of enamel, substances that alter its structural components, type of material used for bonding, and technique employed [2].

The dental enamel should be healthy to permit bonding of brackets; however, dental caries and erosion are common factors that cause loss of mineral components of teeth [2]. Dental caries involves the loss of mineral structure by chemical dissolution due to a reduction in dental biofilm pH [3]. Dental erosion is defined as the induced loss of minerals by acidic substances of nonbacterial origin in contact with the tooth structure [4].

Diets rich in carbonated beverages, fruits, and other acids are being consumed more frequently, which consequently has been increasing the dental erosion [5]. The excess ingestion of these substances is of major concern not only because of high sugar levels, but also because they present pH levels below the critical limit for enamel demineralization (pH < 5.5) [6]. Studies on acidic beverages have demonstrated that these substances cause enamel decalcification around the brackets, consequently increasing the risk of marginal leakage [2, 5].

One of the treatment options to avoid mineral loss is the use of substances with high fluoride concentration, including varnishes and dentifrices. High fluoride dentifrices (above 5000 *μ*g F^−^/g) have been developed for “high risk individuals” [7]. Its efficiency to avoid mineral loss has been confirmed in previous studies [8–10].

However, other studies have demonstrated that the use of fluoridated solutions negatively interferes with the bond strength of orthodontic brackets [11, 12].

This study evaluated the effects of regular and high fluoride dentifrices on the bond strength of brackets to enamel submitted to acid challenge. The null hypotheses tested were as follows: (i) the bond strength of brackets is not affected by acid challenge; (ii) the type of dentifrice does not influence the bond strength of orthodontic brackets.

## 2. Methodology

### 2.1. Preparation of Specimens

Eighty-four permanent bovine incisors were collected and their crowns were separated from the roots, cleaned with periodontal curettes, and stored in distilled water for a maximum period of six months at a temperature of 5°C. The procedures were performed following the specific protocol TR 11405 established by the International Organization for Standardization (ISO) [13]. The crowns were embedded in chemically cured acrylic resin (Jet Clássico, São Paulo, Brazil) in PVC molds (20 mm diameter, PVC Amanco, Joinville, Brazil), maintaining the lingual aspects immersed.

The buccal aspects of crowns were cleaned with fluoride-free prophylactic paste (Dentsply, Konstanz, Germany) for 10 seconds and rinsed with water for the same period.

The 84 specimens were randomly assigned to seven groups (*n* = 12), as described in Table 1.

### 2.2. Dentifrice Treatment

The specimens were immersed in dentifrice (dilution: 3 g of dentifrice/10 mL of distilled water, adding up to 153 g of dentifrice/510 mL of distilled water) for 3 minutes at controlled temperature and pH under constant shaking, using a magnetic shaker (IKA Laboratory Equipment, Staufen im Breisgau, Germany). However, specimens of WN, W3C, and W6C groups were immersed in 600 mL of distilled water under the same conditions of dentifrice treatment.

The treatment cycles were conducted for 7 days, twice a day. After treatment, the specimens were carefully rinsed with distilled water.

### 2.3. Application of Brackets

Metallic brackets for maxillary central incisors (Morelli, Sorocaba, Brazil) with base area of 14 mm^2^ were placed on enamel surfaces of all specimens. The buccal aspect of crowns was conditioned with 35% phosphoric acid (Ultradent, South Jordan, USA) for 20 seconds, rinsed with water, and air-dried. The primer of Transbond XT (Unitek, Landsberg, Germany) was applied following the manufacturer's instructions. Then, the Transbond XT adhesive (Unitek, Landsberg, Germany) was applied on the bracket base, the assembly was placed on the buccal aspect of the crown and a standardized force of 500 g was applied. The excess material was removed with a dental probe (Duflex, Juiz de Fora, Brazil).

A single operator performed all procedures. Each bracket was light cured at a distance of 1 mm from the bracket base to the light-curing tip for 40 seconds, being 10 seconds on each side of the bracket. The specimens were then stored in distilled water (37°C, 24 hours).

### 2.4. Procedures for Dental Erosion (Intervals of Acid Challenges)

The specimens were suspended in 1 L beaker containing 600 mL of orange juice (Del Valle, Santa Bárbara D'Oeste, Brazil) (pH 3.5 ± 0.03) using plastic rods. The orange juice was gently shaken using a magnetic shaker for 15 minutes. The specimens were removed from the orange juice and carefully rinsed with 15 mL of distilled water, removing the acid excess from the surface. In WN group, the specimens were kept in 600 mL of water under 3 minutes of constant shaking.

The acid cycles were performed for 7 days. Twelve specimens in each group were exposed to 3 cycles per day and the other half of specimens were exposed to 6 cycles of acid challenge per day (15 minutes for each cycle). The specimens were stored in artificial saliva during rest. Among cycles, specimens were kept in artificial saliva for 2 hours.

### 2.5. Overnight Storage

The specimens were stored in artificial saliva at controlled temperature and pH. The artificial saliva was prepared as follows: 0.5 mmol/L Ca(NO_3_)_2_ 4H_2_O; 0.9 mmol/L Na_2_HPO_4_ 2H_2_O; 150 mmol/L KCl; 0.02 mol/L H_2_NC(CH_2_OH)_3_ (TRIS); 0.05 *μ*g/mL NaF, pH 7.0 [14, 15].

### 2.6. Bond Strength Test (Shear Bond Strength: SBS)

For the bond strength test, an occlusogingival force was applied by the mechanical testing machine on the upper surface of the bracket between the upper wings and the brackets base, at a speed of 0.5 mm/min [16, 17]. The force required to displace the bracket was measured in Newton (N) and the shear bond strength (SBS) was calculated by dividing the force value by the bracket base area (1 MPa = 1 N/mm^2^).

### 2.7. Analysis of Adhesive Bonded to the Tooth after Debonding of Brackets

**Figure 1:**
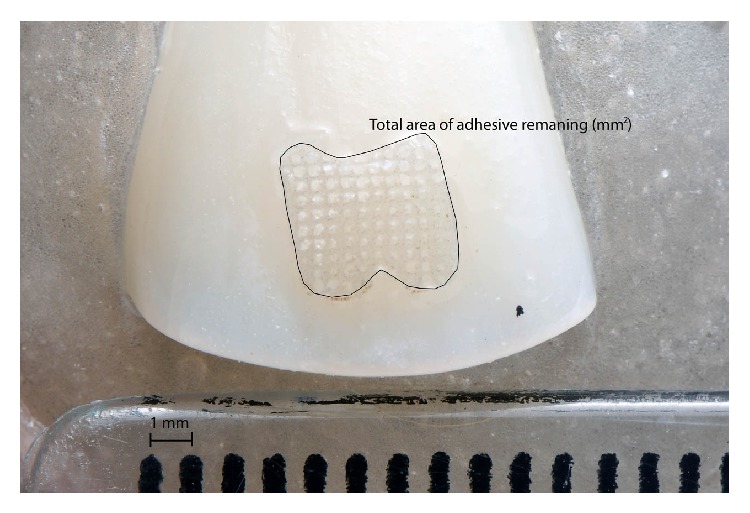
Photograph for analysis of the total area of adhesive bonded to the tooth after debonding of the bracket. Note that a scale was used to serve as reference for the digital scale. Thereafter, the area was calculated on the software Adobe Photoshop CS5.

**Figure 2:**
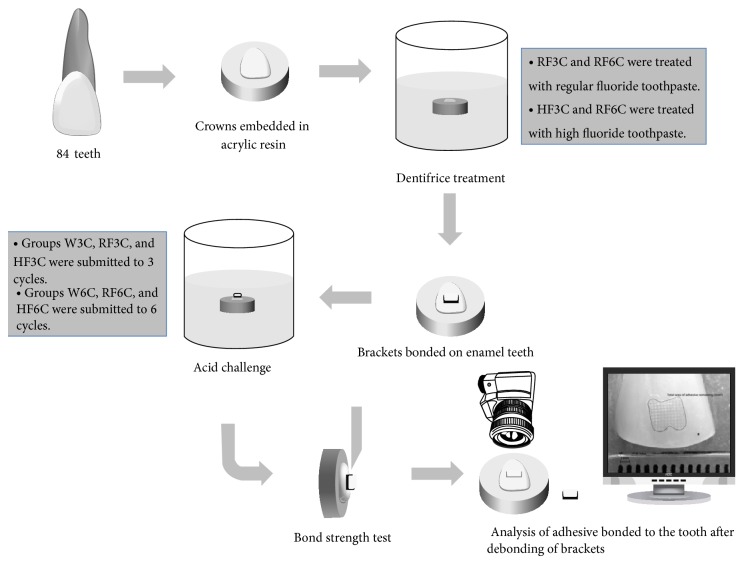
Schematic drawing of the methodology used in this study.

After the shear bond strength, the specimens were photographed with a digital camera (Nikon, Tokyo, Japan) connected to a 100 mm lens (Nikon, Tokyo, Japan). A calibrated ruler was used in the photograph to be used as proportional scale. Thereafter, the area of adhesive bonded to the tooth was calculated on the software Adobe Photoshop CS5 (Adobe Systems Incorporated, San Francisco, USA) (Figure 1).  Figure 2 shows the schematic drawing of the methodology used in this study.

### 2.8. Statistical Analysis

The Shapiro-Wilk normality test and Levene homogeneity test were applied for bond strength tests and area of adhesive remaining data. Bond strength data showed normal distribution and they were analyzed by one-way ANOVA and post-hoc Tukey tests (*P* < 0.05). The area of adhesive remaining did not pass the normality test and was submitted to the Kruskal-Wallis and post-hoc Dunn's tests (*P* < 0.05). The Graph Prism software (Graphpad, La Jolla, USA) was used for statistical analyses.

## 3. Results

**Figure 3:**
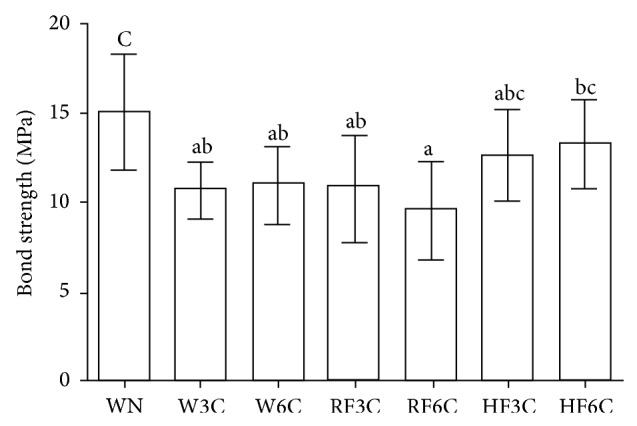
Bond strength values of groups submitted to the shear bond strength test. Different letters indicate statistical difference (one-way ANOVA and post-hoc Tukey tests, *P* < 0.05).

**Figure 4:**
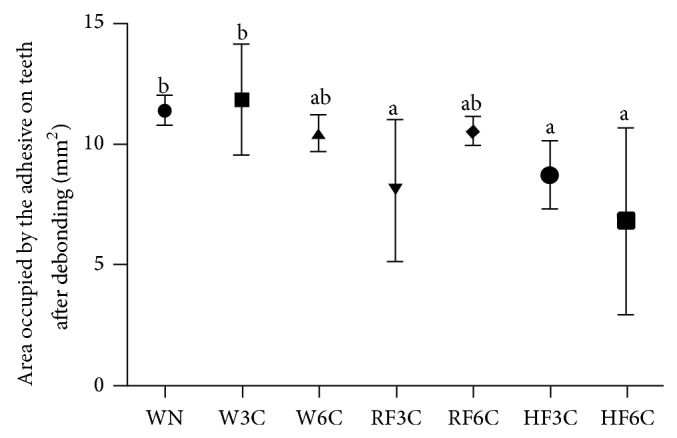
Values in mm^2^ of adhesive material bonded to the tooth after debonding of brackets. Different letters indicate statistical difference (Kruskal-Wallis and post-hoc Dunn's tests, *P* < 0.05).

The means and standard deviations are presented in Figure 3. Groups W3C, RF3C, and HF3C showed no statistically significant differences (*P* > 0.05). Statistically significant difference was found between the regular and high fluoride dentifrices after 6 cycles of acid challenge (*P* < 0.05). The group WN had greater bond strength values than groups W3C, W6C, RF3C, and RF6C (*P* < 0.05). Similar areas of adhesive remaining were found among control groups (WN, W3C, and W6C) and among groups W6C, RF3C, RF6C, HF3C, and HF6C (Figure 4). Additionally, all groups, except group HF6C (6.84 mm^2^), presented mean above 50% (7 mm^2^) of adhesive bonded to the tooth after debonding of brackets.

## 4. Discussion

This study investigated the effects of regular and high fluoride dentifrices on the bond strength of brackets after acid challenge. The results of this study rejected the null hypotheses as the bond strength of brackets is not affected by acid challenge and the type of dentifrice does not influence the bond strength of orthodontic brackets.

This type of dentifrices application and acid challenge has been effective in* in vitro *studies [2, 5, 7, 18]. This investigation evidenced that group WN presented higher bond strength values after the shear bond strength testing than groups W3C and W6C. Previous studies have demonstrated similar characteristic during evaluation of brackets debonding between bovine and human enamel [19–21]. Due to the easy achievement, these teeth may be better selected, increasing the homogeneity of specimens and allowing results with lower method error [11].

The sustained force of 500 g was applied on the bracket to avoid interference in the outcome of bond strength. Studies [22–24] have shown that the no application of sustained force during the bonding process affects the adhesive layer and decreases the bond strength. It happens mainly because the sustained force reduces fluid interference from the underlying tooth. In the present study, the force gauge instrument (Correx Co, Bern, Switzerland) was positioned perpendicularly to the buccal aspect of the crown.

The treatment was performed before bonding of brackets to evaluate if the dentifrices, especially with high fluoride concentration, interfere with the bond strength of brackets in patients presenting dental erosion. Additionally, this sequence of the methodology was made to simulate the regular use of these dentifrices. If the treatment was performed after bonding of brackets, this study did not confirm the second null hypothesis. Therefore, the present results revealed that the high fluoride dentifrice did not negatively interfere with the bonding of brackets, corroborating previous studies using substances with high fluoride concentrations before bonding of brackets [12, 25–27].

Other earlier studies demonstrated that substances with high fluoride concentration might interfere negatively with bonding [28–32]. The application of topical fluoride interferes on enamel etching with phosphoric acid, making it more resistant and reducing its surface energy [29, 30, 32]. Thus, enamel demineralization occurs in a nonstandardized manner, impairing the penetration of adhesive and formation of resin tags [30, 32]. Additionally, no previous study has analyzed the bond strength of adhesive materials using previous treatment with this dentifrice. Notwithstanding the high fluoride concentration, the dentifrice was unable to change the demineralization pattern of phosphoric acid. Flury et al. concluded that fluoride mouthrinses increase the bond strength of composite resin in teeth submitted to dental erosion [33].

Previous studies have demonstrated the efficacy of high fluoride dentifrices to prevent tooth demineralization, acting by the deposition of components, especially fluoride particles, and remineralization of the affected substrate [7, 34]. The present study demonstrated that this dentifrice was able to prevent the reduction of bond strength of brackets submitted to acid challenge. This may be explained by the fact that fluoride particles avoided the enamel demineralization by replacement of calcium and phosphate around the bracket base, thus reducing the chances of premature debonding [33].

Dentifrices with 1450 *μ*g F^−^/g presented similar outcomes with groups W3C and W6C. It may be inferred that regular fluoride concentration was not enough to have a significant influence on reduction of bond strength of brackets. This confirms that high fluoride concentration (5000 *μ*g F^−^/g) was able to remineralize the enamel around the bracket, avoiding the premature debonding.

Many studies employ the Adhesive Remnant Index to evaluate the type of failure occurring after the shear bond strength testing [12, 26, 35, 36]. Even though this method is widely used, it is not able to accurately demonstrate the quantity of adhesive material bonded to the tooth. Therefore, this study used photographs of specimens after debonding of brackets and the area of adhesive material bonded to the tooth was calculated with the aid of a guide ruler on the software Adobe Photoshop CS5. These results demonstrated that, in most specimens, the adhesive material bonded on the tooth was greater than 7 mm^2^. This reveals that, even though enamel demineralization impaired the bonding of brackets, failures substantially occur at the interface between bracket and adhesive material. Also, excessive bonding of bracket is not interesting because this bracket must be removed later, and a strong bonding may impair its removal and cause enamel cracks [37].

## 5. Conclusion

The acid challenge provides significantly lower bond strength values compared to control group (no acid challenge). The high fluoride dentifrice was able to prevent better the reduction in bond strength values of brackets than regular fluoride dentifrices after 6 cycles of acid challenge.

## Figures and Tables

**Table 1:** The groups were divided according to treatment and acid challenge.

Group	Treatment	Toothpaste	Acid challenge
WN	Distilled water		No
W3C			3 cycles
W6C			6 cycles

RF3C	Regular fluoride toothpaste	Colgate Tripla Ação dentifrice, 450 *μ*g F^−^/g, Colgate Palmolive, São Bernardo do Campo, Brazil	3 cycles
RF6C			6 cycles

HF3C	High fluoride toothpaste	Duraphat dentifrice, 5000 *μ*g F^−^/g, Colgate Palmolive, Piscataway, USA	3 cycles
HF6C			6 cycles
